# Addressing the unmet needs of transitional care in juvenile idiopathic arthritis

**DOI:** 10.1093/rheumatology/kead518

**Published:** 2023-09-29

**Authors:** Nihal Şahin, Hafize Emine Sönmez, Nuray Aktay Ayaz, Betül Sözeri

**Affiliations:** Department of Pediatric Rheumatology, Kocaeli University, Faculty of Medicine, Kocaeli, Turkey; Department of Pediatric Rheumatology, Kocaeli University, Faculty of Medicine, Kocaeli, Turkey; Department of Pediatric Rheumatology, Istanbul University, Faculty of Medicine, Istanbul, Turkey; Department of Pediatric Rheumatology, University of Health Sciences, Ümraniye Research and Training Hospital, Istanbul, Turkey

**Keywords:** adult, children, chronic disease, juvenile idiopathic arthritis, rheumatic diseases, transitional care

## Abstract

**Objectives:**

We aimed to comprehensively analyse the available literature to identify the unmet requirements in transitional programs tailored specifically for patients diagnosed with JIA.

**Methods:**

According to published guidance on narrative reviews, a systematic review of the literature on transitional care in rheumatology was conducted. Pertinent documents were collected from reputable databases, such as Web of Science, Scopus, and MEDLINE/PubMed. The search encompassed literature published from the inception of each database until January 2023.

**Results:**

In this study, a comprehensive analysis of the findings of 34 studies was conducted. Among these, 12 studies focused on assessing the readiness of adolescents and young adults diagnosed with JIA. Additionally, 18 studies examined the effectiveness of structured transition programs in terms of adherence and satisfaction. Finally, 4 studies investigated disease-related outcomes in this population.

**Conclusion:**

The need for transitioning children with rheumatic diseases to adult rheumatology services for continued care is clearly evident. However, the absence of established best practice guidelines presents a challenge in facilitating this transition effectively. Although several scoring systems have been proposed to ensure organized and seamless transfers, a consensus has not yet been reached. Furthermore, the socio-economic and cultural variations across countries further complicate the development of universal guidelines for transitioning children with rheumatic diseases. To address these concerns, our objective in conducting this literature review was to emphasize the significance of this issue and identify the specific requirements based on the unmet needs in the transition process.

Rheumatology key messages

- Transitional care has emerged as a new field within the realm of paediatric rheumatology.
- There are many unmet needs in the transition process for patients with juvenile idiopathic arthritis.
- This review highlights major concerns about the assessment of readiness for transition, and the structure of the current transitioning programs, and their effectiveness.

## Introduction

JIA is the most common form of chronic arthritis during childhood, with an incidence of 1:10 000 [1]. Due to advances in the treatment of paediatric rheumatic diseases, an increasing number of children reach adulthood and are destined to transition from the paediatric to the adult health-care system [2]. Although several patients with JIA experience remission, 30–60% of patients continue to have active disease throughout adulthood [3–6]. Long-term follow-up of 246 adult patients with JIA revealed that 43% of patients remained clinically active after an average disease duration of 28 years [7]. Another study showed that 60% of patients with JIA followed through a transitional-care program remained clinically active during adulthood [8].

As children grow up, they need to gain autonomy, and this also applies to children who are dealing with chronic rheumatic diseases. They have to take responsibility for their health. Consequently, transitional care has emerged as a new field in paediatric rheumatology. Although the significance of transitional care has been endorsed in the literature for about 40 years [9], many needs remain unmet regarding its place and convenience in rheumatology practice. As responsibility shifts from parents/caregivers to young adults themselves, the transition process should support the self-management skills of young adults beyond their medical transfer [10]. The Society of Adolescent Medicine defined the transition as ‘a purposeful and planned process for adolescents and young adults (AYAs) with a chronic disease while moving from child-centered to adult-oriented health care systems’ [11]. It is therefore very important to prepare AYAs for transition before they reach the age of 18.

Transition, a pivotal facet within the subjects of paediatric rheumatology, demands the comprehensive attention of clinicians [12]. In 2017, a group of experts convened to establish recommendations for transitional care of AYAs with juvenile-onset rheumatic diseases [13]. Ultimately, they introduced 12 specific recommendations for transitional care [13]. These recommendations primarily focus on establishing a coordinated and timely network between paediatric and adult care settings. Unfortunately, in many countries, there is no standardized approach to the transition process. A recent survey conducted among European paediatric rheumatologists revealed that less than one-third of respondents had a written transition policy [14].

The American Academy of Pediatrics has identified six core elements aimed at enhancing the transition process. An effective transition program should comprise the following six steps: (1) transition policy, (2) tracking and monitoring, (3) transition readiness, (4) transition planning, (5) transfer of care, and (6) transition completion. The initial step of the transition process involves the development of a transition policy. This policy should encompass the approach to transition taken by both paediatric and adult rheumatologists, educate all staff members about this approach, and clearly define the roles of the youth, parent/caregiver, and the paediatric and adult health-care teams involved in the transition process. The second step is to establish criteria and processes for identifying transition-aged youth. The third step of transition is to determine whether or not they are ready for transition. The last three steps are to ensure the transition occurs in a planned and structured way [15]. Even though all the steps have been described in detail in theory, examples of transitional care working smoothly are lacking in practice. Consequently, our aim was to thoroughly examine the existing literature and identify the unmet needs in transitional programs specifically designed for patients with JIA.

### Search strategy

**Figure 1.**
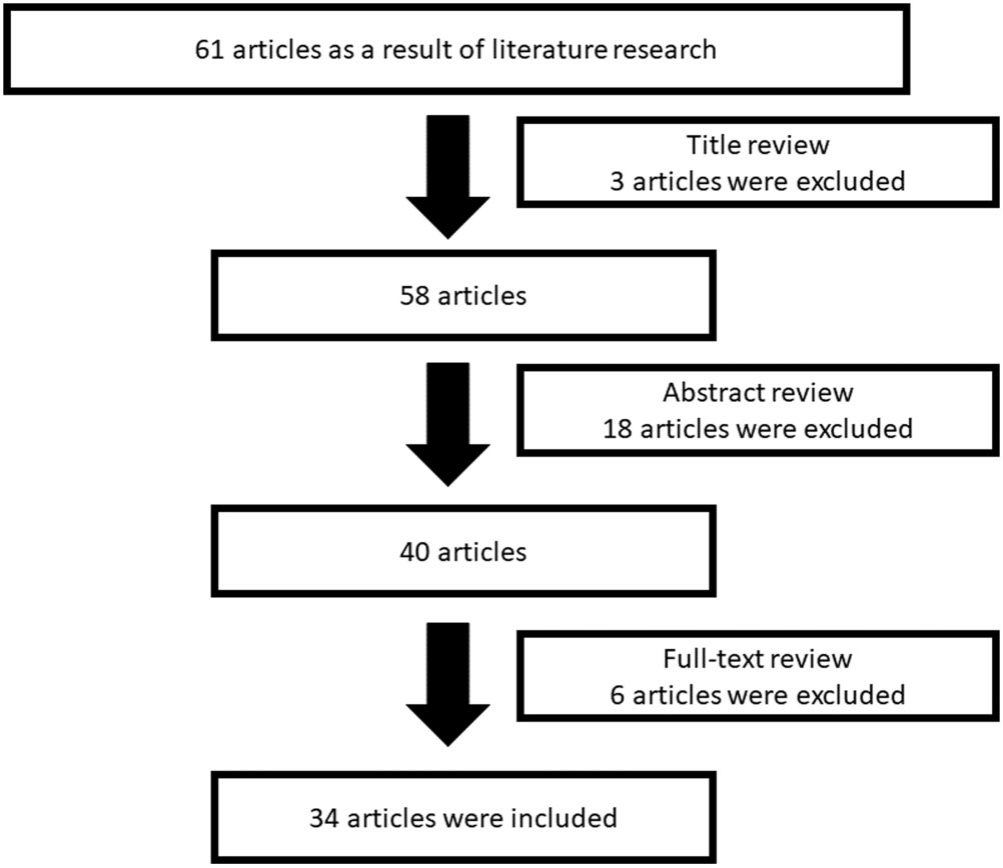
The literature search and study selection process

The literature on transitional care in rheumatology was reviewed. Relevant documents were retrieved from the Web of Science, Scopus, and MEDLINE/PubMed databases according to the published guidance on narrative reviews (Fig. 1) [16]. The literature from the inception of each database to January 2023 was searched using the following keywords: ‘Juvenile Idiopathic Arthritis’ OR ‘Juvenile Rheumatoid Arthritis’ OR ‘Juvenile Chronic Arthritis’ AND ‘transitional care program’ OR ‘transition to adult care’ OR ‘transition care’ OR ‘transition program’.

Articles containing information about readiness for transition of AYAs with JIA, a structured transition program, and its impact on adherence, satisfaction and disease-related outcomes were included, while articles comprising information on transitional care in other rheumatic diseases were excluded. The search was restricted to English-language articles.

### Study characteristics

A total of 34 studies were evaluated. Of them, 12 were related to assessing the readiness of AYAs with JIA, 18 described a structured transition program and its impact on adherence and satisfaction, and 4 discussed disease-related outcomes (Table 1 [15–48]).

**Table 1.** Characteristics of the 34 studies reviewed

Authors	Country	Method	Identification of the participants	Investigated subjects
**Assessment of transition readiness**				
Shaw *et al.* (2005) [15]	UK	Cross-sectional study	308 patients with JIA aged 11 years: *n* = 103; 14 years: *n* = 128; 17 years: *n* = 77	To assess independent consultation, self-treatment, and professional readiness of patients, according to their ages
Currie *et al.* (2022) [16]	Canada	PaCER method (set, collect, and reflect)	9 patients with JIA aged 18–28 years, currently in the process of transfer or having transferred; only 1 participant had formal transition care	Preparedness for transition Continuity and breadth of care Need for supports Ongoing needs
Walter *et al.* (2017) [17]	Netherlands	Survey analysis	48 patients aged 14–16 years with rheumatic diseases (JIA: *n* = 35) and their parents	Patients’ self-management skills
Lazerevic *et al.* (2022) [18]	Serbia	Cross-sectional study	44 patients with JIA and their parents	Relationships between patients’ characteristics and total TRAQ score
Kittivisuit *et al.* (2021) [19]	Thailand	Cross-sectional study	111 patients aged 15–20 years with rheumatic diseases (JIA: *n* = 54)	Conducting a cross-cultural adaptation of TRAQ into the Thai language Assessment of transfer readiness and predictive factors for better readiness
Sonmez *et al.* (2020) [20]	Turkey	Cross-sectional study	157 patients older than 12 years with rheumatic diseases (JIA: *n* = 52) Following up at least six months	Evaluation of transfer readiness and factors affecting readiness
Lazaroff *et al.* (2019) [21]	USA	Cross-sectional study	91 patients aged 17–21 years with rheumatic diseases (JIA: *n* = 64) Their 54 parents	Factors affecting transition readiness in patients with rheumatic diseases
McColl *et al.* (2021) [22]	Canada	Survey analysis, cross-sectional study	71 patients aged 14–20 years with JIA or SLE (JIA: *n* = 61)	Determination of the association between age and sex and transition readiness
Howsley *et al.* (2022) [23]	UK	Correlational cross-sectional study	40 pre-transfer patients aged 10–16 years; 40 post-transfer patients aged 16–24 years, all with JIA Parents of pre-transfer patients	Relationships between psychological and social factors, disease activity, and transfer readiness
Lawson *et al.* (2011) [24]	USA	Cross-sectional study	52 patients aged 13–20 years with rheumatic diseases who would require transition to adult rheumatology care (JIA: *n* = 23)	Health-related self-management before the transition
Roberts *et al.* (2021) [25]	USA	Survey analysis Cross-sectional study	78 patients aged 16 and older with rheumatic diseases (JIA: *n* = 52)	Independent predictors of transition readiness determined by using ADAPT survey
Spiegel *et al.* (2021) [26]	Canada	A phased approach (three phases)	Phase 1A: 19 clinical experts, 2 young adults with JIA, and 1 parent Phase 1B: 15 clinical experts and 30 adolescents with JIA Phase 2: 96 English-speaking and 26 French-speaking patients with JIA	RACER validation in JIA
**Structured transition program and its impact on adherence and satisfaction**				
Akre *et al.* (2018) [27]	Germany, France, Switzerland	Delphi-like study	In the first step: 2 paediatric rheumatologists, 1 adult rheumatologist, 1 transition nurse, 1 family doctor, 1 paediatrician In the second step: 47 international rheumatology experts In the third step: 12 health professionals In the fourth step: 33 international rheumatology experts	Three phases and five age groups were defined in transition. A preparation phase divided into two age groups (12–14 years and 14–16 years) A transfer phase divided into two age groups (16–18 years and 17–22 years) An engagement phase for patients aged 20–24 years Items that reached 80% agreement were defined for each phase .
Shaw *et al.* (2004) [28]	UK	Delphi study	20 patients aged 12–25 years with JIA, 19 parents, and 43 health professionals	Investigation of best practice of and feasibility for the transition program
Hilderson *et al.* (2010) [29]	Belgium	Cross-sectional study	44 patients older than 15 years of age with JIA and already transferred to adult care	Medical follow-up status and health-related outcomes after leaving paediatric rheumatology care
Hilderson *et al.* (2013) [30]	Belgium	Thematic analysis	11 young adults with JIA	The three themes were preparation for transfer, parental involvement, and an adapted adolescent setting The experiences and expectations of the patients were evaluated for each theme
Hilderson *et al.* (2013) [31]	Belgium	Brief of the study design	NA	The content of a brief intervention transition program designed for young people with JIA The rationale and design of a mixed methods study evaluating the clinical impact of the transition program
Hilderson *et al.* (2016) [32]	Belgium	A mixed study that consisted of a quantitative study (longitudinal and comparative analysis) and a qualitative study	In the quantitative longitudinal and comparative study, 27 patients aged 14–16 years with JIA and their parents In the qualitative study, 23 patients aged 17–23 years with JIA who had been transferred without a transition program, and their parents	Effects of the transition program on quality of life and disease-related outcomes
McDonagh *et al.* (2000) [33]	UK	Survey analysis	55 doctors, including paediatric and adult rheumatologists	Understanding current conditions in rheumatology services for AYAs in the transition process and identifying transition needs
McDonagh *et al.* (2004) [34]	UK	Survey analysis	263 health professionals in transitional care for adolescents with JIA	Health professionals’ perception of education and training involved in transitional care for adolescents with JIA
Shaw *et al.* (2004) [35]	UK	Focus group	12 patients aged 11–18 years and 18 patients aged 19–30 years with JIA, and 23 of their parents	Patients’ (and their parents’) perspectives on their transition program
McDonagh *et al.* (2006) [36]	UK	Survey analysis	308 patients aged 11–17 years with JIA, and 303 of their parents	To develop an evidence-based transitional care program for adolescents with JIA To evaluate the acceptability and utility of the program resources
McDonagh *et al.* (2007) [37]	UK	Cross-sectional study	308 patients aged 11–17 years with JIA, and 303 of their parents	To evaluate the effect of an evidence-based transitional care program on adolescents’ HRQL
Shaw *et al.* (2007) [38]	UK	A phased approach (three phases)	308 patients aged 11–17 years with JIA, and 303 of their parents	To develop a scale for use in assessing satisfaction with transitional health care among adolescents with a chronic illness, and among their parents Preliminary validation of this scale
Shaw *et al.* (2007) [39]	UK	Survey analysis	308 patients aged 11–17 years with JIA, and 303 of their parents	Opinions of adolescents with JIA (and of their parents) about the quality of their transitional care
Jensen *et al.* (2015) [40]	USA	Cross-sectional study	236 patients aged 16 years and older with rheumatic diseases in transition services (JIA: *n* = 112)	Outcome of and satisfaction with individualized transition programs
Overbury *et al.* (2021) [41]	USA	Observational cross-sectional study	57 patients seen in the transition clinic in 2 years (JIA: *n* = 36)	Completion of the transition program and the rate of patients continuing care in adult rheumatology clinics
Boeker *et al.* (2022) [42]	Germany	Observational cross-sectional study	85 patients with rheumatic diseases (JIA: *n* = 60) who met these criteria: the follow-up duration was at least 2 years; they attended at least two visits to the Tuebingen Transition Program; they attended at least one visit at the age of 17–18 years	Outcomes of the Tuebingen Transition Program: HRQL, EQ-5D-5L, disease activity, and physical activity, and comparison with the German population
Shaw *et al.* (2004) [43]	UK	Survey analysis	478 health professionals	Essential points in transition care for patients with JIA
Anelli *et al.* (2017) [44]	Brazil	Survey analysis	112 certified paediatric rheumatologists registered with the Brazilian Society of Rheumatology	National transitional practice in paediatric rheumatology centres
**Disease-related outcomes**				
Mannion *et al.* (2016) [45]	USA	Retrospective cohort study	58 patients with JIA aged >14 years who had a follow-up duration encompassing the period at least 6 months before the last paediatric rheumatology visit to at least 6 months after the first adult rheumatology visit	Transition interval length, changing treatment and diagnosis code, and admission to physical therapy or emergency room after the transition
Relas *et al.* (2018) [46]	Finland	Cross-sectional study	Two cohorts included 214 JIA patients aged 16–20 years who visited the Helsinki University transition clinic The first cohort: Nov 2012 – May 2013 The second cohort: Apr 2015 – Apr 2016	No differences between the two cohorts (regarding the disease status of the patients in the transition process) were found.
Mikola *et al.* (2022) [47]	Denmark, Finland, Norway, Sweden	Prospective cohort study	408 patients with JIA in an 18-year follow-up study Directly transferred (*n* = 163), later referred (*n* = 50) or not transferred (*n* = 195)	Transition rate Disease activity status after the transition
van Pelt *et al.* (2018) [48]	Netherlands	Prospective cohort study	215 patients with JIA	Effect on disease activity of dropout in transition care, and factors affecting dropout in transition care

ADAPT: ADolescent Assessment of Preparation for Transition, AYAs: Adolescents and Young Adults, HRQL: Health-Related Quality of Life, EQ-5D-5L: the 5-level EuroQol-5D, PaCER: the Patient and Community Engaged Research, RACER: Readiness for Adult Care in Rheumatology, TRAQ: Transition Readiness Assessment Questionnaire.

### Assessment of readiness of patients and their parents for transition

Providing transitional care at the correct time is quite important. However, the best time for transition has not yet been determined. During the early discussions on the concept of transition in JIA, the evaluation of transitional readiness was based on independent consultations, self-medication, and vocational readiness across three age groups: 11, 14 and 17 years. A study by Shaw *et al.* [17] found that those patients aged 17 had better readiness according these measures. In a patient-led qualitative study by Currie *et al.* [49], all nine participants with JIA emphasized the importance of readiness as a crucial component in the transition process. They reported that their paediatric rheumatologists supported them in developing self-advocacy skills and preparedness, but they were not formally assessed regarding their transition readiness. Walter *et al.* [50] evaluated transfer readiness and self-management skills in AYA aged 14–20 years with rheumatic diseases, including 72% with JIA, and found that only half of them had received information about the transition.

In order for the transition to work properly, it is necessary to measure whether AYAs are ready or not for transition. Many tools have been published that are designed for use in assessing readiness to transition. However, there is no standard tool. Studies have been conducted using various tools, such as the Transition Readiness Assessment Questionnaire (TRAQ) [51], TRANSITION-Q [52], the Am I ON Taking Responsibility for Adolescent/Adult Care (ON TRAC) [53], the University of North Carolina (UNC) TRANSITION scale [54], the Self-Management and Transition to Adulthood with Rx = Treatment (STARx) questionnaire [18], and the Good2Go questionnaire [19]. The most preferred tool in rheumatology practice is the TRAQ, consisting of 20 items divided into 2 domains: self-management and self-advocacy [51]. The answers are scored by using a 5-point Likert-type scale. Higher scores indicate greater readiness for transitional care. However, there is no cut-off value indicating whether the patient is ready for transition or not. In our literature review, four articles using TRAQ were found [20–23]. The TRAQ score was strongly correlated between patients with JIA and their parents [20]. Female gender, older age, and improved health behaviours were found to be related to higher TRAQ scores. Furthermore, higher health literacy of parents/caregivers increased TRAQ scores of AYAs, although this increase was statistically insignificant [23]. Sonmez *et al.* [22] evaluated the transition readiness of patients older than 12 (one-third with JIA) and their parents. All patients were inactive, and TRAQ scores did not differ between patients and parents. The self-management score was higher in females than in males. The TRAQ score was not affected by the diagnosis of the patient. A study evaluating only JIA patients showed no significant relationship between patient characteristics such as sex, disease subtypes, disease activity, and TRAQ scores [20]. The Thai version of TRAQ showed excellent internal consistency and good-to-excellent reliability in Thai patients with rheumatic diseases. About half of the patients had JIA. Questions about health insurance coverage, financial management, and financial support (question 15) had the lowest scores. Independent visits were the only significant independent predictors of a high TRAQ score. Dependent visits predicted low TRAQ scores in appointment keeping, and inactive disease status predicted low TRAQ scores in health tracking issues [21].

The Transition-Q questionnaire is another validated tool utilized to evaluate the self-management skills of adolescents with chronic health conditions, providing an estimate of their readiness to transition. The questionnaire comprises 14 items arranged in increasing order of difficulty, and respondents aged 14–20 years chose one of three responses: ‘never’, ‘sometimes’, or ‘always’. The scores are calculated out of 100, with higher scores indicating better proficiency in self-management skills. In a study of 70 patients aged 14–20 years, 90% of whom had JIA, the mean total score was 59 for patients with JIA; 83% of patients responded ‘never’ to the item ‘I go to the doctor’s appointment alone.’ A positive correlation was detected between age and the total Transition-Q score. After controlling for age and disease duration, no significant gender differences were observed in Transition-Q scores [24, 52].

Howsley *et al.* [25] conducted a study to explore the psychological and social factors that influence the readiness to transition of pre- and post-transfer AYAs with JIA and their parents. To measure transition readiness, they employed two assessment tools—the Ready Steady Go/Hello to Adult Services (RSG/HTAS) and the Self-Efficacy for Managing Chronic Disease 6-item Scale. The RSG/HTAS tool assessed transfer-related knowledge and skills, while the Self-Efficacy for Managing Chronic Disease 6-item Scale measured health-related self-efficacy. The study found that the parents had higher transfer-related knowledge and skills than the patients. Additionally, post-transfer AYAs with low disease activity scores had better transfer-related knowledge and skills, and self-efficacy. The study also found a significant correlation between better transfer-related knowledge and skills, lower generalized anxiety in pre-transfer AYAs, and lower depression levels in both pre-and post-transfer AYAs. Lastly, post-transfer AYAs with low generalized anxiety had better self-efficacy [25].

The tool used to assess independence in self-management tasks among adolescents with chronic illness was developed as a crucial component of the transition process. This tool consisted of 15 questions categorized into four domains related to medication management, medical appointments, health insurance and information management, and other health-care skills. The questionnaire was administered to patients between the ages of 13 and 20 years, with 44% of them having a diagnosis of JIA. The findings indicated that approximately half of the participants were knowledgeable about medication names, purposes, and side effects, and were able to take their medications as prescribed. However, a significant number of adolescents were unable to independently arrange medical appointments. Additionally, only a small portion of them had thoughts or knowledge about health insurance and information management [26]. These results highlight areas where further support and education may be necessary to enhance the self-management skills of adolescents with chronic illnesses, including those diagnosed with JIA.

The Adolescent Assessment of Preparation for Transition (ADAPT) survey is an additional tool available for assessing the readiness of AYAs aged 16 years or older with rheumatic diseases for transition. The ADAPT survey consists of three domains: self-management, prescription medications, and transfer planning. According to the survey results, most participants reported receiving guidance on managing their health and taking medications. However, less than half of the participants received detailed information about the transition process itself. Notably, patients below the age of 19 obtained lower total scores compared with older patients, indicating a potential need for additional support and preparation. Additionally, participants with lower levels of education achieved lower scores across all three domains compared with those with higher levels of education [55]. This suggests that education level may influence the readiness and knowledge of AYAs for transition.

The tools mentioned earlier for assessing transition readiness are generally designed to evaluate readiness across various chronic diseases and may not fully address the specific needs and challenges faced by patients with JIA. This is particularly relevant as chronic joint problems associated with JIA can potentially hinder patients from assuming responsibility for their own care or acting independently. Given these unique circumstances, it may be beneficial to develop or adapt assessment tools that specifically address the challenges and expectations of individuals with JIA. Most recently, Spiegel *et al.* [27] developed a new tool called RACER (Readiness for Adult Care in Rheumatology) to assess the transition instrument in youth with JIA. This new measurement consists of 32 items, including the following domains: (1) general knowledge (8 items), (2) knowledge about medications (3 items), (3) planning for adult life (5 items), (4) managing your health condition (6 items), (5) standing up for yourself (6-items), and (6) knowing how to get around the health-care system (4-items). The focus point of the RACER is solely on patients with JIA. Apart from general knowledge, all domains had good internal consistency in JIA patients. Moreover, strong or moderate correlations were shown among all domains. When the baseline and 2-week-later test scores were evaluated, strong test–retest reliability was found [27]. The RACER had similar or better internal consistency than other validated transition readiness questionnaires, such as the TRAQ ‘Self-Management’ subscale and the ‘Self-Advocacy’ subscale [28].

### Unmet needs in the assessment of readiness

There are currently several unmet needs in the field of transitional care (Table 2). For instance, validation studies of the available questionnaires are lacking for each language and for diverse cultural contexts. Even though many questionnaires exist to assess readiness, no comparative analysis of these evaluations has been conducted on patients with JIA. Thus, it remains unclear which questionnaire is the most appropriate one for JIA. The domains of the questionnaires have varying degrees of significance in assessing transition readiness in JIA, and the optimal cut-off values for such domains remain unknown. Deciding that patients are ready, based on a questionnaire result, cannot predict that the transition will work properly in real life. Many unpredictable factors may influence the process. For instance, the recently developed system for assessing transition readiness in JIA patients does not take into account the impact of joint-related limitations on self-management tasks. Taking into account the unique challenges posed by joint-related limitations, it is crucial to have assessment tools that comprehensively and specifically assess the readiness of JIA patients for transition to adult care. Such tools may provide a more accurate and specific assessment of readiness, allowing health-care professionals to better support and guide JIA patients during the transition.

**Table 2.** The unmet needs in transition care for JIA patients

**Unmet needs in the assessment of readiness**
Invalid questionnaires for each language and culture for assessment of transition readiness Lack of a cut-off for current questionnaires that determines whether patients are truly ready to transition Insufficiency of current questionnaires to measure the effects of specific conditions associated with the disease on transition readiness Insufficiency of current transfer programs to meet the needs of patients diagnosed during adolescence
**Unmet needs in the structure of a transition program**
Insufficiency of current transition programs to cover the different needs associated with different socio economic conditions, health-care systems, and insurance structures across different countries Lack of data on transition in developing countries Inadequate resources to finance such programs Difficulties in adapting programs in developed countries for use in low-income countries Lack of awareness of the importance of continuation of health care, which results in underestimation of the need for non-emergency health services such as transition Lack of continuation of health insurance for patients Lack of access to the adult centre to which the patient has been transferred, due to a change in their place of residence for education or work
**Unmet needs in assessment of outcome**
Changing of the diagnosis of patients in adult outpatient clinics Insufficiency of current registry systems to measure the outcomes for patients in the long term

Another issue is that up to one-third of JIA presents during adolescence [17]. It may be quite difficult to prepare AYAs for transition shortly after the diagnosis. The transition program may be ignored by both clinicians and patients while the patient is still adapting to many new conditions, such as the presence of chronic disease and medications.

### Structured transition program and its impact on adherence and satisfaction

A coordinated and structured transitional care program is necessary for the continuity of health care, and some Delphi studies have been conducted to establish an organized transition program [29, 30]. However, only a very limited number of studies report on structured transition programs. Clemente *et al.* [31] underlined the insufficiency of structures, staffing, and processes of transition care in rheumatic diseases. They concluded that a structured transition program is required. Hilderson *et al.* [32] assessed the adherence to the adult care setting of 44 JIA patients transferred without an organized program. Of these 44 patients, only 25 reported that an adult rheumatologist had followed them up. The remaining patients did not continue to their regular follow-up (*n* = 13) or were followed by their general practitioner (*n* = 6). Another study by the same group [33] investigated the experiences and expectations of AYAs with JIA who had been transferred to adult care without a planned transition process. This group of patients reported that sudden encounters with elderly patients with severe RA were a rather bad experience, and they reported the need for an adaptive approach. The authors concluded that preparation of a structured transition program is essential. Subsequently, the same group [34] designed a transition program called the DON’T RETARD (Devices for the Optimization of Transfer and Transition of Adolescents with Rheumatic Disorders) project. This program consisted of the following five steps: (1) first outpatient appointment with a transition coordinator (TC); (2) second outpatient appointment with TC; (3) information day for adolescents and their parents; (4) individualized transfer plan; and (5) actual transfer. They described this project as a brief program and suggested that it may be more feasible in clinical practice. The clinical impact of the DON’T RETARD project on transition was evaluated in another study [35]. In that study, 27 patients who participated in this project were compared with 45 patients who directly transferred to adult care. The study confirmed that this brief transition program improved rheumatic-specific health status and quality of life.

At the beginning of the 20th century, the British Paediatric Rheumatology Group recognized the importance of transitional care, and they conducted survey studies to measure health professionals’ knowledge on this subject [36, 37]. They also questioned AYAs with JIA about their perspective on transition [38]. The adolescents confirmed the necessity of an individualized structured transition program. Afterward, they designed a coordinated transitional care program based on their obtained information [39]. They suggested an individualized transition plan through the coordination of a local health caregiver and a consultant rheumatologist. According to the aforementioned program, adolescents should be tested for their self-completion and knowledge, skills, and awareness of resources during each visit, and their development stages should be reviewed every 6 months. After announcing this structured transitional care program, the same group evaluated the program’s impact on health-related quality of life in patients with JIA [40]. They found a significant improvement in health-related quality of life in the presence of a structured transitional care program. Subsequently, the same group developed and validated a scale called ‘Mind the Gap’ to assess satisfaction with transition care among AYAs with JIA [41]. The scale questioned the perceptions of AYAs. According to this scale, patients were considered dissatisfied if their perceptions of ‘current care’ were rated less than their expectations of ‘best care’. After initial validation, this scale was tested on patients with JIA who were enrolled in a structured and coordinated transitional program [42]. They showed that a structured and coordinated transitional program improved the overall satisfaction of both patients and their parents.

Jensen *et al.* [56] managed the transition process with the help of a social worker. Initially, a social worker handed patients and their parents a workbook describing the process. After completing the workbook, the social worker documented the transition goals of both the patients and their parents. In the follow-up, the social worker followed up on the problems of the patients and their parents regarding transitional care and arranged an appointment with an adult rheumatologist when the transitional age came. After the initial scheduled adult rheumatology appointment, the social worker contacted patients to ask how many times the patient had visited the adult rheumatologist. Despite such a structured program, only 42% of the patients experienced a successful transition. Furthermore, 10% of the patients did not visit an adult rheumatologist after the initial scheduled appointment, and 15% of the patients had never seen an adult rheumatologist and continued to be followed up in paediatric care. Many factors influence the satisfaction of the transition program, but adherence is one of the indispensable factors. In the same report, the authors evaluated the patients’ satisfaction with the transition program and reported that most of the patients were satisfied with the transition process. However, they were able to get feedback from only 27% of the patients who had transitioned to adult service [56].

Overbury *et al.* [43] designed a transition clinic named ACCORD (the Adult Center for Childhood-Onset Rheumatic Disease), which took place once a week in the paediatric rheumatology clinic, accompanied by a staffed adult rheumatologist. Patients were referred to this clinic at the age of 16 years or older. The clinic visits in ACCORD were performed without parents, to enable AYAs to gain responsibility. The number of visits was individualized according to patient needs. The process followed the six core elements [15]. Another transition program, called the Tuebingen Transition Program, was announced by Boeker and colleagues [44]. This program usually started at age 13 years, depending on the maturity and disease activity of the patient. During the visits, the AYAs were assessed regarding their knowledge of their diseases and therapies. The visits continued until AYAs gained their autonomy.

Walter *et al.* [45, 50] reported on a new transition program from the Netherlands. In this program, an individual transition plan was designed for each patient. AYAs with JIA who were aged 12–23 years were examined in a dedicated adolescent JIA clinic by a health-care team consisting of a mixture of paediatric- and adult-care health professionals. They reported that this transition pathway achieved high satisfaction and a low drop-out rate for care.

### Unmet needs in the structure of a transition program

Implementing an individualized transition program for patients with JIA is imperative to ensure continuity of follow-up care and prevent discontinuity in disease management. Critical components of such a program include fostering patient independence and introducing patients to the adult clinic environment and rheumatology health professionals in the pre-transition phase, as well as post-transition monitoring of follow-up care by health-care providers. Developing an optimal transition program is challenged by the varying socio-economic conditions, health-care systems, and insurance structures across different countries.

Most studies on transition have been conducted in developed countries, and the data on transition care in developing countries is very scarce. Even in developed countries, inadequate resources have been reported as the main barrier to providing a transition program [46]. The socio-cultural status and development level of the country may influence the functioning of health care. A survey from Brazil showed that only 13% of paediatric rheumatology centres had a well-established transition program [47]. The aforementioned study concluded that the current poor economic situation of developing countries might cause clinicians to underestimate the importance of non-emergency health services, such as transition. Since individuals in developing countries have to face particular problems, such as lack of health insurance, and unemployment, special programs are required for these countries.

Furthermore, when the patient turns 18, he/she can change city or even country to find a job or go to university and may encounter a health-care provider who does not know him/her at all. Moreover, in some countries, the expiration of health insurance in the transition from childhood to adulthood may make it difficult to access a doctor.

### Disease-related outcomes

There are significant differences between paediatric and adult rheumatologists in terms of both treatment approaches and diagnoses. For instance, Mannion *et al.* [48] reported that the diagnosis of adult patients with JIA was more likely to change to RA in adult care.

Relas *et al.* [57] reported that a coordinated transition period could achieve stable disease activity and high clinical attendance rates. However, a limited number of studies focus on the disease-related outcomes of AYAs who are transferred to adult care. Mikola *et al.* [58] reported that patients who were directly transferred to the adult clinic at the age of 18 years, had significantly higher active joints and disease activity scores compared with those who were transferred to the adult clinic later. Despite a structured transition program, almost a quarter of patients (22%) dropped out of follow-up during the transition process [59]. Low disease activity during the transition process was identified as a risk factor for the failure of the transition [59].

## Conclusion

In providing the continuation of health services for JIA patients who have reached adult age, paediatric rheumatologists may benefit from the experience of other disciplines in which the need for transitional care has been identified longer. As type 1 diabetes is one of the most common chronic diseases worldwide, many studies have been conducted about the transitional care of patients with type 1 diabetes. Recently, Sanmugalingham *et al.* [60] announced a project called ‘Keeping in Touch’, which delivers tailored transition support. They hypothesized that using a reminder mobile phone application may improve adherence to the transition process. Similar applications may be used in paediatric rheumatology. As the use of social media continues to grow, it is now possible to connect with patients through these platforms. Sharing information between rheumatology experts and patient groups via social media during the transition period may help patients stick to their treatment plans better. This approach can also make health-care providers more aware of any issues they might not have noticed before.

In conclusion, it is evident that a significant number of children with rheumatic diseases require transition to adult rheumatology centres. However, there is a lack of established best practice guidelines for facilitating this transition. In consideration of the intricate interplay between socio-economics and cultural dynamics, each nation has to create its own program. By conducting a literature review, our aims were to highlight the importance of this issue and to identify the specific requirements based on the unmet needs of the transition process. This thorough review of the relevant literature may provide the necessary foundation for creating a standardized program that will suit each country’s specific needs.

## Data Availability

No new data were generated or analysed in support of this research.
